# Successful treatment of angioinvasive aspergillosis causing diaphragmatic rupture with bowel perforation and cerebral aspergillosis in a patient with *FLT3*-mutated acute myeloid leukemia: A case report

**DOI:** 10.1097/MD.0000000000028700

**Published:** 2022-01-28

**Authors:** Nan Young Bae, Ja Min Byun, Chang Kyung Kang, Pyoeng Gyun Choe, Nam Joong Kim, Min-Sung Kim, Kyu Joo Park, Sung-Soo Yoon

**Affiliations:** a Department of Internal Medicine, Seoul National University Hospital, Seoul, Korea; b Department of Neurosurgery, Seoul National University Hospital, Seoul, Korea; c Department of Surgery, Seoul National University Hospital, Seoul, Korea.

**Keywords:** acute myeloid leukemia, case report, cerebral aspergillosis, disseminated aspergillosis

## Abstract

**Rationale:**

Throughout the clinical course of acute myeloid leukemia (AML), aspergillosis infection remains a significant determinant of treatment outcomes and survival. To emphasize the importance of early diagnosis and appropriate application of integrated therapeutic approaches, we present a case of AML patient who survived through angioinvasive aspergillosis infection causing diaphragmatic rupture with bowel perforation and cerebral aspergillosis during active AML treatment.

**Patient concerns:**

A 39-year old male with *FLT3*-mutated AML was transferred to our hospital due to persistent fever after induction therapy.

**Diagnosis and interventions:**

During voriconazole treatment for his invasive pulmonary aspergillosis, the patient was diagnosed with colon perforation at splenic flexure and suspected perforation of left diaphragm with communication with left pleural space. Although pancytopenic, emergency laparotomy was performed with granulocyte transfusion. Also, dual antifungal therapy with voriconazole and micafungin was applied. With supportive care, he was able to successfully complete 3 cycles of consolidation using tyrosine kinase inhibitor. However, 80 days after the last chemotherapy, the patient experienced seizure caused by a single 1.5 cm sized enhancing mass in the right occipital lobe. Diagnostic and therapeutic mass removal was carried out, and pathology-confirmed cerebral aspergillosis was diagnosed.

**Outcomes:**

The patient's neurologic symptoms are resolved and he is leukemia free, but remains on voriconazole for his cerebral aspergillosis till this day.

**Conclusions:**

Our case highlights the importance of timely integrated intervention and adequate underlying disease control in treatment of invasive aspergillosis in immunocompromised patients. Such rigorous efforts can save even the most seemingly dismal case.

## 1 Introduction

Throughout the clinical course of acute myeloid leukemia (AML), infectious complications remain a significant determinant of treatment outcomes and survival. Invasive aspergillosis remains a major clinical issue in AML despite the introduction of posaconazole prophylaxis,^[1,2]^ with a 10%-incidence during induction or consolidation neutropenia^[3,4]^ and aspergillosis-attributable mortality rate ranging around 30% to 40%.^[5,6]^ The first step to optimal management of invasive aspergillosis is early diagnosis and early initiation of adequate antifungal therapy.^[7]^ In addition to antifungal therapy, surgery should be considered for certain patients, especially in those complex cases with chronic necrotizing disease.^[8]^ The decision to perform surgery depends on numerous factors, including the location of the lesions, the extent of resection required, performance status, underlying disease, and comorbidities. Patients with AML rarely represent appropriate candidates for surgery due to postsurgical complications related to cytopenia.

Reduction of immunosuppression is another important component of invasive aspergillosis management.^[7]^ The worst outcomes occur in patients with persistent severe immune dysfunction, and in those with organ dysfunctions that limit administration of therapeutic dose of antifungals. Although the relative contribution of these prognostic indicators is unclear, it is generally accepted that in chemotherapy-induced neutropenic patients, recovery of bone marrow function is critical to the control of aspergillosis.^[9]^

Here, we present a case of a 39-year old male patient with AML who survived through angioinvasive aspergillosis infection causing diaphragmatic rupture with bowel perforation and cerebral aspergillosis during active AML treatment.

## 2 Case presentation

**Figure 1:**
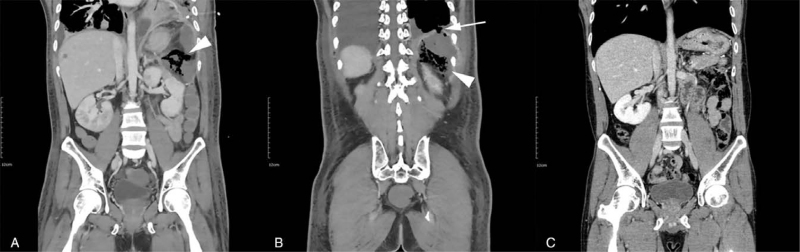
(A), (B) Abdominal CT scan shows perforation of colon at splenic flexure with extraluminal air content (arrowhead) and suspected perforation of left diaphragm with communication with left pleural space (arrow). (C) About 13 mo after emergency laparotomy, abdominal CT scan shows disappeared air bubbles and nearly resolved fluid cavity in left subdiaphragmatic space. CT = computed tomography.

**Figure 2:**
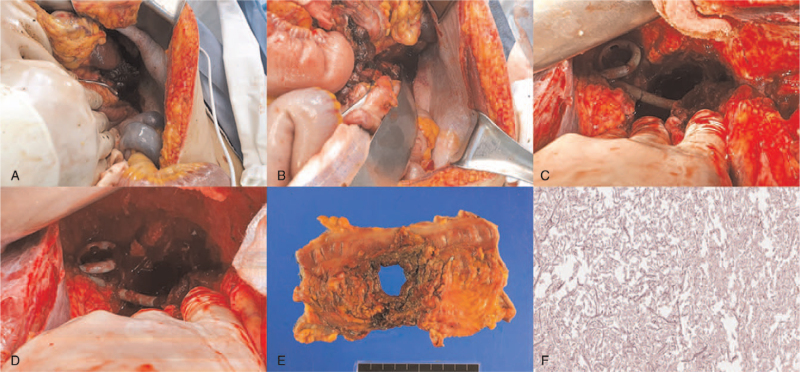
(A), (B) Intraoperative view of emergency laparotomy shows splenic flexure perforation with subphrenic necrotic tissue and diaphragm perforation. (C), (D) Intraoperative view of colostomy repair shows remnant abscess cavity in subphrenic space. (E) Gross surgical specimen obtained after segmental resection of colon shows perforation of the splenic flexure. (F) Debridement tissue pathology shows numerous fungal hyphae and spores consistent with aspergillus species (H & E stain, ×40).

A 39-year old male with *FLT3*-ITD mutated AML was referred to our hospital for a second opinion after an attempt to induce remission with standard 7 + 3 cytarabine/idaraubicin regimen. Upon admission to our hospital, the patient was pancytopenic (white blood count or WBC 0.09×10^3^/μL, hemoglobin or Hb 9.2 g/dL, platelet 23×10^3^/μL, absolute neutrophil count or ANC 0/μL) even though 38 days had passed since induction chemotherapy. The patient had already received vancomycin, meropenem and amphotericin B liposome (3 mg/kg/d) for 11 days prior to transfer to control neutropenic fever, but since he had fever of 38.9 °C upon admission, blood culture and computed tomography (CT) of chest and abdomen were performed along with galactomannan assay, sputum tuberculosis polymerase chain reaction (PCR), sputum *Pneumocystis jirovecii* PCR and blood cytomegalovirus PCR. The sputum tuberculosis PCR, sputum *P jirovecii* PCR and blood cytomegalovirus PCR were negative, but the galactomannan antigen assay was positive (index 5.00). The chest CT scan showed multiple pulmonary nodules with central necrosis and peripheral ground glass opacities. The abdominal CT scan showed infarction at spleen, distal pancreas, and left kidney. Neutropenic condition, lung lesions with a halo sign and cavity, and the positive result in the galactomannan antigen assay supported a diagnosis of invasive aspergillosis. As results, probable invasive pulmonary aspergillosis and vancomycin-resistant enterococci bacteremia was diagnosed and the patient was put on voriconazole, linezolid plus piperacillin-tazobactam. Meanwhile, bone marrow (BM) examination was also done to assess leukemic burden and the results revealed morphologic leukemia-free state and normal karyotype but *FLT3*-ITD positive (variant to wild type allele ratio 111/3562, 0.03). After weighing up pros and cons, granulocyte colony-stimulating factor was initiated for infection control. The patient seemed to stabilize with such measures, but 12 days into the treatment the patient complained of severe abdominal pain. Upon physical examination, muscle guarding was noted thus another CT scan of abdomen was ordered: the CT scan showed colon perforation at splenic flexure and suspected perforation of left diaphragm with communication with left pleural space, on top of multi-focal liver abscesses (Fig. 1A, B). The patient was still pancytopenic (WBC 0.11×10^3^/μL, Hb 8.9 g/dL, platelet 18×10^3^/μL, ANC 67/μL), but operation was arranged. At the same time, granulocyte transfusions as adjunctive treatment were performed for invasive aspergillosis infection control. Emergency laparotomy revealed proximal descending colon perforation, localized abscess on left subphrenic area penetrating left diaphragm, and necrotic changes of spleen, kidney, and peritoneum. Colon segmental resection with double-barrel colostomy, primary repair of diaphragm with omental flap interposition for patch to subphrenic space, and debridement of necrotic tissue was performed (Fig. 2). Also, dual antifungal therapy with micafungin plus voriconazole was initiated because of progress to severe infection despite voriconazole monotherapy. Another reason of dual antifungal therapy was because intra-abdominal candidiasis could not be completely ruled out considering the extent, severity and clinical characteristics of the infection. Fortunately, the patient quickly recovered after the surgery and by postoperative day 4 he was able to eat and by postoperative day 6 his ANC recovered to 5544/μL.

By this point, it had been 55 days since 7 + 3 induction therapy. Based on his *FLT3*-ITD positivity, high dose cytarabine (HiDAC) plus midostaurin consolidation was planned. However, the patient was deemed unfit for cytotoxic chemotherapy, thus only midostaurin (50 mg twice a day for 14 days) was administered. Follow-up *FLT3*-ITD was negative. Second consolidation therapy was carried out 46 days after the abdominal surgery with HiDAC plus midostaurin. The patient suffered from disseminated zoster infection during second consolidation, which was controlled with intravenous acyclovir (10 mg/kg/dose every 8 hours). Due to formation of new skin lesions and slowly resolving lesions, intravenous acyclovir was given for about 3 weeks. BM examination after second consolidation showed complete remission (CR) with negative *FLT3*-ITD. The patient was on dual antifungal therapy with micafungin plus voriconazole throughout the 2 cycles of consolidation. After extensive discussion with the patient, allogeneic hematopoietic stem cell transplantation was deferred until CR2, and the patient underwent third consolidation with HiDAC plus midostaurin 91 days after the abdominal surgery. Final BM examination after the third consolidation showed CR with negative *FLT3*-ITD. Voriconazole was continued throughout the whole process with voriconazole therapeutic drug monitoring to check whether or not the drug level is appropriate every 3 to 4 weeks.

**Figure 3:**
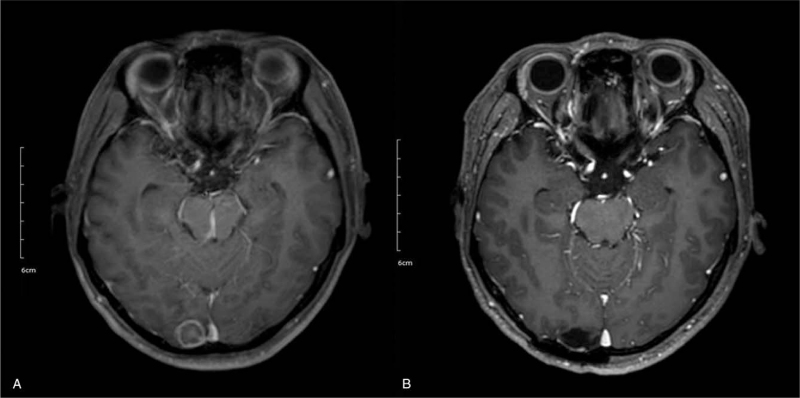
(A) Brain MRI shows 1.5 cm sized rim enhancing mass in the right occipital lobe. (B) About 5 mo after mass removal, brain MRI shows no definite residual lesion in the right occipital lobe. MRI = magnetic resonance imaging.

**Figure 4:**
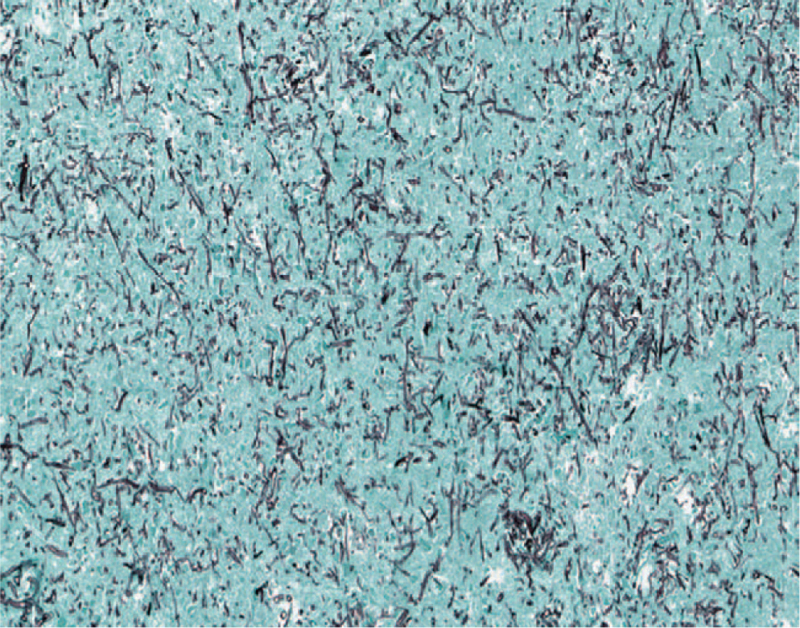
Removed brain tissue pathology shows multiple thin, septate hyphae consistent with aspergillus species (Grocott methenamine silver stain, ×40).

Eventually, the patient was able to return to work and was only occasionally followed-up to check voriconazole therapeutic drug monitoring, hemogram and *FLT3*-ITD status. Unfortunately, he suffered a generalized tonic-clonic seizure 80 days after the last chemotherapy (171 days after initial abdominal surgery; 220 days after induction chemotherapy). Brain magnetic resonance imaging revealed a single 1.5 cm sized enhancing mass in the right occipital lobe (Fig. 3A). BM re-examination showed remained CR. Thus, diagnostic and therapeutic mass removal was planned and the patient underwent craniotomy with mass removal. Grocott methenamine silver stain was used on the removed tissue from the right occipital lobe and the surgical pathology was confirmed to be aspergillosis fungal ball with central necrosis and fibrous wall (Fig. 4). The patient's neurologic symptoms are resolved and he is leukemia free, but remains on voriconazole for his cerebral aspergillosis till this day.

## 3 Discussion

Despite recent advances in antifungal therapy, invasive fungal infections still remain a leading cause of morbidity and mortality in immunocompromised patients, especially in those with hematologic malignancies and profound neutropenia. In patients with AML who received intensive chemotherapy, the overall incidence of encountering invasive fungal infections was 16.9% and among them, mold infections caused by aspergillus species were about 60%.^[3]^ The 2016 Infectious Diseases Society of America guideline suggests voriconazole monotherapy as the initial treatment of choice for invasive aspergillosis.^[7]^ Because mortality of invasive aspergillosis is high in spite of voriconazole treatment, there were the studies for combined triazole and echinocandin therapy^[10]^ in order to exploit potential synergies and a broader spectrum activity and to prevent resistance.^[11]^ However, because of the variable test designs and conflicting results of preclinical testing about combination therapy, primary dual antifungal therapy is not routinely recommended.^[7]^ However, the Infectious Diseases Society of America guidelines recommends to consider dual antifungal therapy with voriconazole and an echinocandin for severe disease in select patients,^[7]^ including those with underlying hematologic malignancies and/or profound prolonged neutropenia. Accordingly, our patient was treated with dual antifungal agents, micafungin plus voriconazole, based on the extent of his infection and benefited from such aggressive measures.

On top of adequate antifungal therapy, combination of different modalities such as surgery should also be considered in some cases. Surgical interventions are usually implemented to control localized aspergillosis that is accessible to debridement or complications of invasive aspergillosis.^[7]^ In our case, as the patient was already diagnosed with invasive pulmonary aspergillosis, gastrointestinal aspergillosis was highly suspected. Due to colon perforation surgical intervention was inevitable and with the help of experienced surgeons, the patient came through and the surgical debridement greatly expedited infection control (Fig. 1C). Also, when he unexpectedly came to the hospital due to generalized tonic-clonic seizure, we were careful not to rush to the conclusion of extramedullary relapse of AML. Through timely but extensive work-up comprised of PET-CT, brain and spine magnetic resonance imaging, cerebrospinal fluid cytology, BM examination with molecular and genetic studies, and pathologic confirmation, the patient was able to avoid unnecessary treatment. After removal of isolated fungal ball in the brain, the patient remains symptom free till this day (Fig. 3B).

**Figure 5:**
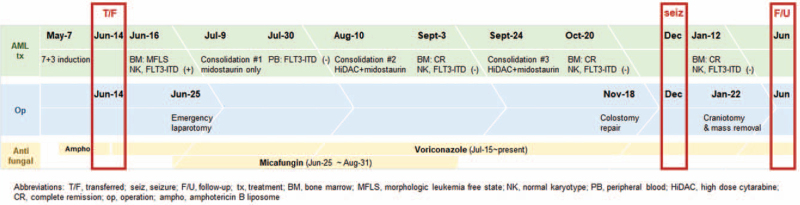
Summary of patient's clinical course.

Another major axis of dealing with invasive aspergillosis infection in neutropenic patients is the recovery of neutropenia itself.^[11]^ Adjunctive measures, namely granulocyte transfusions and granulocyte colony-stimulating factor, can be considered.^[7]^ However, the control of underlying disease to induce bone marrow function recovery is ultimately fundamental in overcoming neutropenia. The AML treatment heavily relies on multiple rounds of cytotoxic chemotherapy and hematopoietic stem cell transplantation.^[12]^ Fortunately, with recent recognition of driver mutations and development of target therapies,^[13]^ we now have more options for AML treatment. Midostaurin, a type I *FLT3* inhibitor, is one of these options. It is known to significantly improve overall survival when added to standard induction and consolidation therapy in *FLT3* mutated AML patients.^[14]^ Since our patient harbored *FLT3*-ITD mutation, midostaurin was used in creative combination with backbone chemotherapy depending on the patient's condition to control AML (Fig. 5). The patient remains in CR state, and hematopoietic stem cell transplantation has been deferred until second CR due to co-morbidities.

## 4 Conclusion

All in all, accurate diagnosis and early initiation of adequate treatment is important for improving survival rates of invasive aspergillosis infection.^[15]^ Therefore, actively diagnostic tests were performed and a wide range of treatment options were considered based on a patient's conditions. Our case demonstrated that timely integrated intervention and adequate underlying disease are critical. Interdisciplinary approaches, including aggressive antifungal therapy, appropriate surgical intervention, and control of underlying disease, can save even the direst cases.

## Acknowledgments

We would like to extend our sincere gratitude and appreciation to all the medical personnel of Seoul National University Hospital involved in taking care of this patient. We would also thank the patient and his family for letting us share his experience.

## Author contributions

Designed the study: JMB. Abdominal surgical intervention: KJP. Neurosurgical intervention: MSK. Infectious disease specialists/advisors: CKK, PGC, NJK. Acquisition of data: NYB, JMB. Wrote the first draft: NYB, JMB. Revised the paper: all the authors. All authors have read and approved the manuscript.

**Conceptualization:** Ja Min Byun.

**Investigation:** Nan Young Bae, Ja Min Byun, Chang Kyung Kang, Pyoeng Gyun Choe, Nam Joong Kim, Sung-Soo Yoon.

**Methodology:** Ja Min Byun.

**Resources:** Nan Young Bae.

**Visualization:** Nan Young Bae, Min-Sung Kim, Kyu Joo Park.

**Writing – original draft:** Nan Young Bae.

**Writing – review & editing:** Nan Young Bae, Ja Min Byun, Chang Kyung Kang, Pyoeng Gyun Choe, Nam Joong Kim, Min-Sung Kim, Kyu Joo Park, Sung-Soo Yoon.
